# Editorial: Optical imaging and manipulation in the biological world

**DOI:** 10.3389/fbioe.2026.1982350

**Published:** 2026-09-08

**Authors:** Xiaoshuai Liu

**Affiliations:** School of Physics and Materials Science, Guangzhou University, Guangzhou, China

**Keywords:** biophotonics applications, cellular microrobot, optical fiber, optical imaging analysis, optical manipulation techniques

The integration of optical imaging and manipulation technologies has fundamentally transformed our capacity to explore and intervene in living systems across scales—from single molecules to entire organisms. Innovations such as super-resolution microscopy, multiphoton imaging, adaptive optics, optical tweezers, optogenetics, and light-mediated gene editing have enabled real-time visualization of cellular dynamics and precise modulation of biological processes, driving breakthroughs in diagnostics, therapeutics, and fundamental life science research. Despite these advances, critical barriers continue to hinder broader adoption and clinical translation. Imaging artifacts in thick scattering tissues, trade-offs between spatial resolution and acquisition speed, invasiveness during prolonged manipulation, and the lack of scalable, cost-effective strategies remain unresolved. Recent progress in AI-driven image analysis, hybrid systems combining optics with microfluidics or spectroscopy, and novel nanomaterials for enhanced light-matter interactions offer promising solutions. This Research Topic was conceived to showcase cutting-edge methodologies that overcome these barriers, emphasizing technical innovation, biological validation, and practical applicability.

Over the course of the call, we received six submissions; five were accepted after rigorous peer review. The published works collectively illustrate how novel fiber probes, multimodal diagnostics, tissue clearing, and light-driven cellular machines are pushing the frontiers of optical bioengineering. Below, we summarize each contribution and highlight the thematic threads that connect them:

Liang et al. developed a molecularly imprinted polymer/CdTe quantum dot-decorated polymer optical fiber microprobe for amoxicillin detection (Liang et al.). The core–shell design achieved a linear detection range of 0.5–50 μg/L with a limit of detection of 0.31 μg/L, alongside excellent specificity, reusability, and biocompatibility. This sensor enables residue-free, *in situ* monitoring, addressing critical needs in pharmaceutical sensing and environmental surveillance.

Xie and Liu introduced a tapered fiber probe (TFP) for non-contact near-infrared optical pacing in zebrafish embryos (Xie and Liu). Achieving 3 µm full-width at half-maximum precision at physiologically relevant working distances, the authors demonstrated developmental-stage-dependent pacing sensitivity and 1.7-fold greater photosensitivity in sinoatrial versus ventricular regions. Calcium imaging confirmed a photothermal mechanism activating thermosensitive channels. This work establishes a proof-of-concept platform for contactless, high-precision cardiac conduction control *in vivo*.

Lindtner et al. combined Raman microscopy with micro-computed tomography to detect staphylococcal bone infections (Lindtner et al.). Raman spectra revealed reduced phosphate, Amide III, and CH_2_ deformation bands in infected bone, with a single principal component explaining 96%–98% of the variance. Micro-CT quantified trabecular deterioration, and combined features improved support vector machine-based pathogen-specific discrimination between *Staphylococcus aureus* and *Staphylococcus epidermidis*. This multimodal approach offers rapid, non-destructive diagnosis for orthopedic applications.

Reedich et al. developed a whole-mount optical clearing and multiplex fluorescence imaging workflow for the rabbit tenuissimus muscle (Reedich et al.). For the first time, they achieved α-bungarotoxin labeling of γ motor endplates compatible with solvent-based clearing, enabling simultaneous visualization of extrafusal and fusimotor terminals. Confocal and light-sheet imaging revealed spindle densities of 1,600 ± 217 spindles/g and enabled quantitative morphometrics of capsule length, volume, and intrafusal fiber numbers, overcoming limitations of conventional spindle histology.

Ma et al. reviewed light-force-powered cellular medical micromachines, covering bacteria, microalgae, red blood cells, immune cells, and subcellular structures (Ma et al.). Optical tweezers enable non-contact, subcellular-precision assembly and control, with applications in targeted drug delivery, minimally invasive surgery, and immunotherapy. The review identifies key challenges—penetration depth, phototoxicity, operation intelligence—and outlines future directions including adaptive optics swarm control and bioprinting integration.

Collectively, these contributions embody the core themes of this Research Topic: fiber-based interfaces bridging free-space optics to biological targets, multimodal integration of molecular and structural signatures, deeper imaging through scattering tissues, and the transition from passive observation to active optical manipulation. They demonstrate that interdisciplinary collaboration—combining photonics, materials science, biology, and computation—can overcome longstanding barriers and accelerate the translation of optical technologies into next-generation biomedical tools. We thank all authors, reviewers, and the editorial team for making this Research Topic a success.

